# TgATAT-Mediated α-Tubulin Acetylation Is Required for Division of the Protozoan Parasite *Toxoplasma gondii*

**DOI:** 10.1128/mSphere.00088-15

**Published:** 2016-01-20

**Authors:** Joseph M. Varberg, Leah R. Padgett, Gustavo Arrizabalaga, William J. Sullivan

**Affiliations:** a Department of Pharmacology and Toxicology, Indiana University School of Medicine, Indianapolis, Indiana, USA; b Department of Microbiology and Immunology, Indiana University School of Medicine, Indianapolis, Indiana, USA; University at Buffalo

**Keywords:** microtubules, cytoskeleton, lysine acetylation, Mec-17, acetyltransferase, endodyogeny

## Abstract

Toxoplasma gondii is a widespread protozoan parasite that causes potentially life-threatening opportunistic disease. New inhibitors of parasite replication are urgently needed, as the current antifolate treatment is also toxic to patients. Microtubules are essential cytoskeletal components that have been selectively targeted in microbial pathogens; further study of tubulin in *Toxoplasma* may reveal novel therapeutic opportunities. It has been noted that α-tubulin acetylation at lysine 40 (K40) is enriched during daughter parasite formation, but the impact of this modification on *Toxoplasma* division and the enzyme mediating its delivery have not been identified. We performed mutational analyses to provide evidence that K40 acetylation stabilizes *Toxoplasma* microtubules and is required for parasite replication. We also show that an unusual *Toxoplasma* homologue of α-tubulin acetyltransferase (TgATAT) is expressed in a cell cycle-regulated manner and that its expression peaks during division. Disruption of *TgATAT* with CRISPR/Cas9 ablates K40 acetylation and induces replication defects; parasites appear to initiate mitosis yet exhibit incomplete or improper nuclear division. Together, these findings establish the importance of tubulin acetylation, exposing a new vulnerability in *Toxoplasma* that could be pharmacologically targeted.

**IMPORTANCE** *Toxoplasma gondii* is an opportunistic parasite that infects at least one-third of the world population. New treatments for the disease (toxoplasmosis) are needed since current drugs are toxic to patients. Microtubules are essential cellular structures built from tubulin that show promise as antimicrobial drug targets. Microtubules can be regulated by chemical modification, such as acetylation on lysine 40 (K40). To determine the role of K40 acetylation in *Toxoplasma* and whether it is a liability to the parasite, we performed mutational analyses of the α-tubulin gene. Our results indicate that parasites cannot survive without K40 acetylation unless microtubules are stabilized with a secondary mutation. Additionally, we identified the parasite enzyme that acetylates α-tubulin (TgATAT). Genetic disruption of *TgATAT* caused severe defects in parasite replication, further highlighting the importance of α-tubulin K40 acetylation in *Toxoplasma* and its promise as a potential new drug target.

## Abstract (precis)

*Toxoplasma gondii* is an opportunistic parasite that infects at least one-third of the world population. New treatments for the disease (toxoplasmosis) are needed since current drugs are toxic to patients. Microtubules are essential cellular structures built from tubulin that show promise as antimicrobial drug targets. Microtubules can be regulated by chemical modification, such as acetylation on lysine 40 (K40). To determine the role of K40 acetylation in *Toxoplasma* and whether it is a liability to the parasite, we performed mutational analyses of the α-tubulin gene. Our results indicate that parasites cannot survive without K40 acetylation unless microtubules are stabilized with a secondary mutation. Additionally, we identified the parasite enzyme that acetylates α-tubulin (TgATAT). Genetic disruption of *TgATAT* caused severe defects in parasite replication, further highlighting the importance of α-tubulin K40 acetylation in *Toxoplasma* and its promise as a potential new drug target.

## INTRODUCTION

*Toxoplasma gondii* is an obligate intracellular protozoan parasite in the phylum *Apicomplexa*, which also includes *Plasmodium* spp. and *Cryptosporidium* spp., the causative agents of malaria and cryptosporidiosis, respectively. *Toxoplasma* is capable of infecting virtually any nucleated cell in warm-blooded animals and is estimated to be present in ~30% of humans worldwide (1). *Toxoplasma* can infect a new host congenitally or following the ingestion of oocysts shed in the feces of cats (the definitive host) or tissue cysts present in undercooked meat (2). While the immune system is capable of controlling the acute infection caused by the rapidly proliferating tachyzoite stage of the parasite, *Toxoplasma* is able to differentiate into a latent bradyzoite stage, establishing a chronic infection that remains for the life of the host. Reactivation of these bradyzoite cysts in immunocompromised individuals can lead to debilitating or life-threatening disease; for example, toxoplasmosis accounts for 10 to 30% of the deaths of HIV/AIDS patients (3, 4) and is a risk factor for patients receiving organ transplants or undergoing immunosuppressive chemotherapies (5–8). The frontline antifolate treatment is effective at targeting the replicative stage, but serious toxicity issues necessitate the identification of better drug targets (9).

Constructed from tubulin monomers, microtubules are a component of the cytoskeleton and are required for a variety of steps in *Toxoplasma* biology, including parasite replication (10). Upon invasion, tachyzoites undergo a form of asexual division termed endodyogeny (11, 12) in which two daughter parasites assemble within the mother parasite. This complex process is dependent upon the function of two distinct microtubule populations (13, 14). The spindle microtubules originate from a microtubule-organizing center (MTOC) termed the centrosome and ensure proper chromosome segregation and karyokinesis as the parasite completes each round of closed (intranuclear) mitosis (13, 14). Progression through mitosis is partnered with expansion of the daughter parasite cytoskeleton driven by elongation of the 22 subpellicular microtubules from a second MTOC, the apical polar ring, toward the basal end of the parasite. Once formed, the subpellicular microtubules are nondynamic, tethered to the cytosolic face of the parasite inner membrane complex (IMC), a collection of flattened vesicles originating from the endoplasmic reticulum-Golgi network (15). In addition to replication, microtubules play critical roles in a variety of aspects of *Toxoplasma* biology, including parasite motility, host cell attachment, and invasion; consequently, microtubules have emerged as attractive targets for therapeutic intervention (10). In addition, the mechanisms by which *Toxoplasma* microtubules are regulated is an active area of inquiry.

Microtubule functions are regulated by a diverse collection of posttranslational modifications (PTMs) that occur on the α- and β-tubulin subunits that make up microtubule polymers (16–19). Most of these tubulin PTMs occur on the C-terminal tails that extend out from the surface of the microtubules to regulate interactions with effector microtubule-associated proteins (20, 21). In contrast, acetylation of lysine 40 (K40) on α-tubulin is a unique PTM that resides within the lumen of microtubules (22, 23). K40 acetylation is well conserved across eukaryotes, and the primary acetyltransferase that delivers this PTM was recently identified as Mec-17/ATAT, a Gcn5-related N-acetyltransferase (24, 25). Originally identified in the flagella of the unicellular green alga *Chlamydomonas reinhardtii* (26, 27), K40 acetylation was subsequently found to be enriched on long-lived microtubules throughout higher eukaryotes (28, 29). Recent studies with *Caenorhabditis elegans* suggest that K40 acetylation stabilizes microtubules by promoting the formation of a salt bridge that augments interactions between α-tubulin subunits in adjacent protofilaments (30). Despite mounting evidence supporting a role for K40 acetylation in microtubule stability, the biological function of this PTM *in vivo* remains unclear, as evidenced by the variety of phenotypes observed across numerous systems when K40 acetylation is manipulated (24, 31–33).

Numerous tubulin PTMs, including acetylation of K40, have been catalogued in *Toxoplasma* (34–37), but the functional importance of these modifications for microtubule dynamics and parasite biology remains largely undefined. In this study, we established the role of α-tubulin K40 acetylation by generating endogenous α-tubulin point mutants and through CRISPR-mediated disruption of the *Toxoplasma* α-tubulin acetyltransferase orthologue (named TgATAT). Together, these approaches reveal that K40 acetylation is critical for the stabilization of tachyzoite microtubules, which is required for daughter cell formation and karyokinesis. The discovery that TgATAT-mediated acetylation of α-tubulin is necessary for *Toxoplasma* replication establishes the importance of tubulin PTMs in apicomplexan parasites.

## RESULTS

### K40 acetylation is dispensable only if tubulin is stabilized through another mutation.

*Toxoplasma* α-tubulin can be acetylated at K40, but the functional significance of this PTM has yet to be addressed. Three α-tubulin isotypes are present in the *Toxoplasma* genome: TGME49_316400 (*TgTUBA1*), TGME49_231770 (putative α-tubulin I), and TGME49_231400 (tubulin/Ftsz family). *TgTUBA1* is the only isotype expressed in tachyzoites and the only one that contains the conserved K40 residue reported as acetylated (34). To address the role of α-tubulin K40 acetylation in tachyzoites, we generated three parasite clones with different K40 mutations in the endogenous *TgTUBA1* genomic locus: (i) lysine to arginine (K40R), which conserves the positive charge of lysine but prevents acetylation, (ii) lysine to glutamine (K40Q), which is an acetyl-lysine mimic (38), and (iii) a silent mutation as a control (K40K). To select for positive clones harboring mutant K40, our construct included a second mutation that confers resistance to the microtubule-disrupting drug oryzalin (39, 40). Since studies with other species suggest that K40 acetylation stabilizes microtubules, we chose to generate oryzalin resistance by mutating valine 252 to lysine (V252L), a mutation in the GTPase-activating domain that provides high levels of oryzalin resistance but has no effect on microtubule stability (41).

**FIG 1:**
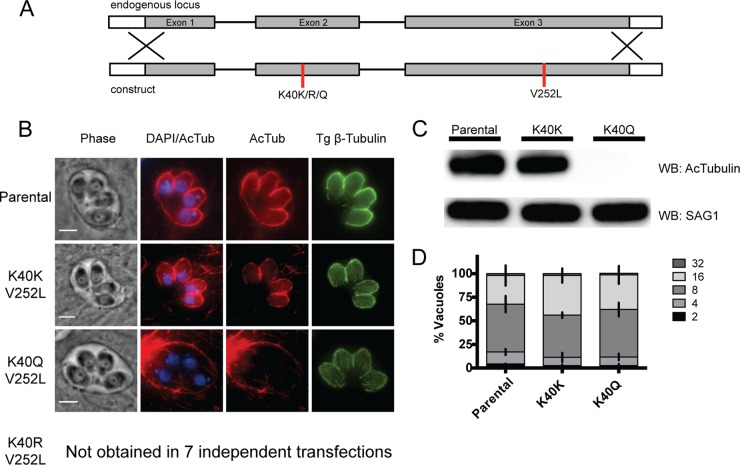
Ablation of K40 acetylation is not tolerated unless it is replaced with the K40Q acetyl-lysine mimic. (A) Diagram of *TgTUBA1* genomic locus aligned with the construct used to replace the endogenous locus by double homologous recombination. The construct contains the nonstabilizing V252L oryzalin resistance mutation and the K40K, K40R, or K40Q mutation. (B) IFAs of K40 mutants stained for acetyl-K40–α-tubulin (red) or β-tubulin (specific to *Toxoplasma*, green). The K40Q mutation results in complete loss of K40 acetylation in the parasite but not its host cell. Images were merged with the DNA stain DAPI (blue). Scale bars, 3 μm. (C) Western blot (WB) assay of parental RH and mutant parasites showing loss of K40 acetylation in K40Q mutants. The blot was probed with anti-acetyl-K40–α-tubulin and anti-SAG1 antibodies as a loading control. (D) Doubling assays performed to assess the growth of parental RH and mutant parasites. Replication rates were determined by counting the parasites within 100 random vacuoles at 24 h postinfection. Three independent trials were conducted, and the average percentage of vacuoles with the indicated number of parasites ± the standard error of the mean is shown (no significant difference between mean percentages of vacuoles at each stage between strains as determined by two-way analysis of variance).

Figure 1A illustrates the allelic replacement strategy used to make K40 mutants by using the nonstabilizing V252L mutation to confer oryzalin resistance. The *TgTUBA1* gene was amplified from genomic DNA isolated from oryzalin-resistant clones, and the presence of the expected mutations in each established line was confirmed by sequencing. While both the K40K and K40Q mutations were readily obtained, K40R mutant parasites were never obtained despite seven independent attempts. The acetylation status of α-tubulin K40 was assessed by using both immunofluorescence assays (IFAs) and Western blotting (Fig. 1B and C). K40Q mutant parasites show a complete loss of K40 acetylation (as expected, the glutamine acetyl-lysine mimic does not cross-react with the anti-K40-acetyl α-tubulin antibody) but no obvious defects in any microtubule structures, as visualized with a *Toxoplasma*-specific β-tubulin antibody (Fig. 1B). It is noted that cross-reactivity of the K40-acetyl antibody used (6-11B-1) results in a signal from the host microtubules outside the parasitophorous vacuole (PV) (for example, Fig. 1B, K40K and K40Q). Host microtubules are actively recruited to the PV shortly after invasion by *Toxoplasma* (42); variability in the enrichment of host microtubules accounts for the variation in the K40-acetyl signal observed in host cells. Parasite growth assays further show that replacement of K40 with the glutamine acetyl-lysine mimic (K40Q) did not affect the replication rate (Fig. 1D).

**FIG 2:**
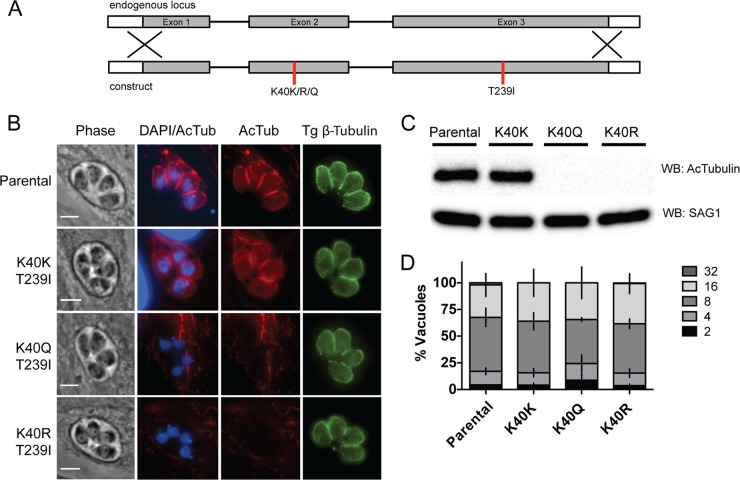
K40 acetylation is dispensable in the presence of the T239I oryzalin resistance mutation. (A) Diagram of the *TgTUBA1* genomic locus aligned with the allelic replacement construct containing the oryzalin resistance mutation T239I and the K40K, K40R, or K40Q mutation. (B) IFAs of mutant parasite lines stained for acetyl-K40–α-tubulin (red) or β-tubulin (green). Images were merged with the DNA stain DAPI (blue). Scale bars, 3 μm. (C) Western blot (WB) assay of parental RH and mutants confirms the loss of K40 acetylation in K40Q and K40R parasites. The blots were probed with anti-acetyl-K40–α-tubulin and anti-SAG1 antibodies as a loading control. (D) Doubling assays performed as described in the legend to Fig. 1D to assess the growth of parental RH and mutant parasites.

Some mutations in the oryzalin binding pocket stabilize microtubules by promoting protofilament interactions and tubulin polymerization (41). Given the reported role of K40 acetylation in microtubule stability, we hypothesized that an oryzalin-resistant mutation in this binding pocket might allow the generation of K40R mutants. We therefore generated constructs containing K40K, K40R, and K40Q by using the T239I oryzalin binding pocket mutation (Fig. 2A), which was chosen for the similarity of its levels of drug resistance to those of V252L (43). Sequencing of the *TgTUBA1* gene confirmed the presence of both mutations in the oryzalin-resistant clones. In the T239I oryzalin-resistant background, all K40 mutants, including K40R, were readily obtained. Both K40Q and K40R parasites showed a complete loss of K40 acetylation, as visualized by both IFA and Western blotting (Fig. 2B and C), yet displayed no difference in replication relative to the silent mutation or the wild type (Fig. 2D).

Our mutational analyses show that ablation of α-tubulin K40 acetylation is possible only when a second, stabilizing mutation (e.g., T239I) is present or when K40 is mutated to a residue (glutamine) that mimics an acetylated lysine. These results suggest that K40 acetylation is required in tachyzoites and likely contributes to the stabilization of microtubules.

### Identification of an acetyltransferase that colocalizes with acetylated tubulin during tachyzoite division.

**FIG 3:**
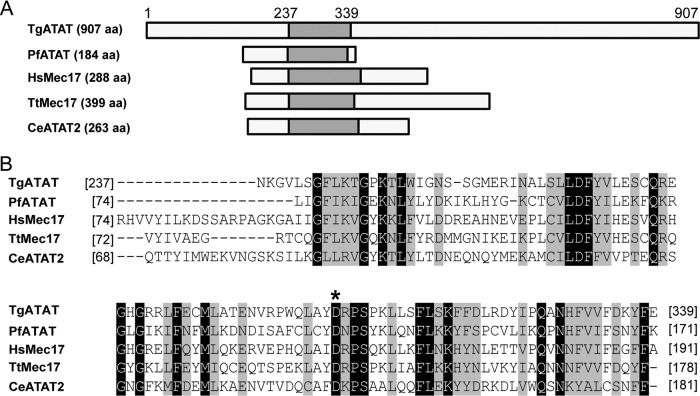
Comparison of ATAT/Mec-17 homologues. (A) Depiction of ATAT protein sequences from *T. gondii* (TgATAT, TGME49_31600), *Plasmodium falciparum* (PfATAT, PF3D7_0924900), *Homo sapiens* (HsMec17, XP_005249477.1), *Tetrahymena thermophila* (TtMec17, TTHERM_00355780), and *C. elegans* (CeATAT2, CELE_W06B11.1), with the number of amino acids (aa) in parentheses. Gray boxes represent the lysine acetyltransferase domain. (B) Amino acid sequence alignment of the KAT domain of the indicated ATAT homologues with identical residues highlighted in black and similar residues highlighted in gray. The asterisk denotes an aspartic acid residue previously shown to be important for ATAT activity (43).

To further characterize the role of α-tubulin K40 acetylation in tachyzoites, we sought to identify the enzyme delivering this PTM. In other species, α-tubulin acetyltransferase (ATAT, also known as Mec-17) has been implicated as the primary enzyme acetylating tubulin (24, 25, 44, 45). A bioinformatic survey of ToxoDB.org v.24 (46) revealed a single gene containing a Mec-17 domain belonging to the Gcn5-related superfamily, TGME49_319600, which we will refer to as *TgATAT*. *TgATAT* is located on chromosome IV and contains two exons encoding a predicted protein of 907 amino acids (Fig. 3A). We aligned the lysine acetyltransferase (KAT) domains of ATAT homologues from several representative species with Clustal Omega (47, 48) (Fig. 3B). Interestingly, while the Mec-17 domain is highly conserved, including the key residues critical for enzymatic activity (Fig. 3B), the predicted TgATAT protein is considerably larger than all previously characterized ATAT/Mec-17 proteins (Fig. 3A) (24, 44). *TgATAT* transcripts are expressed in tachyzoites, peaking in the early stages of mitosis, waning during cytokinesis, and remaining at basal levels during interphase, suggesting that TgATAT is a cell cycle-regulated protein (46, 49).

**FIG 4:**
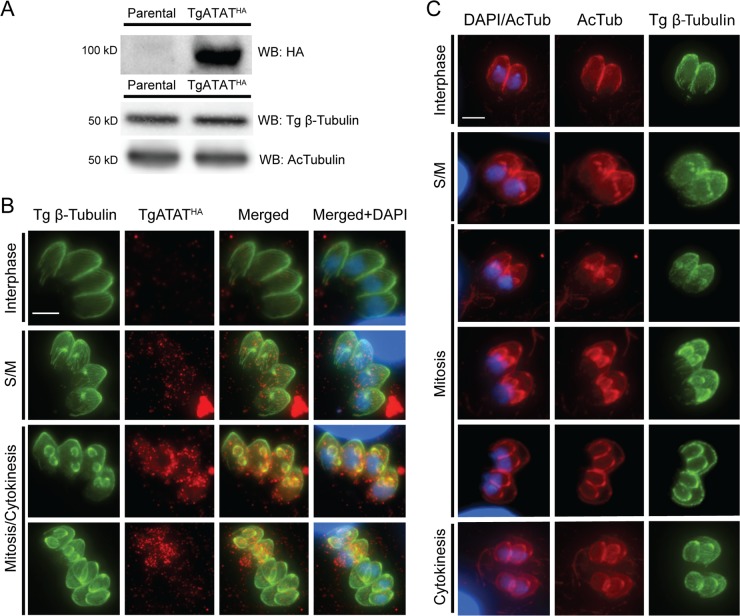
Expression of TgATAT and acetylated α-tubulin during the tachyzoite cell cycle. (A) Western blot (WB) assay of lysates from the parental strain (RHΔ*hx*Δ*ku80*) and parasites containing endogenously HA-tagged TgATAT (TgATAT^HA^). The blot was probed with antibodies recognizing the HA epitope or acetyl-K40–α-tubulin. β-Tubulin was also probed as a loading control. (B) IFAs of TgATAT^HA^ parasites stained for HA (red, TgATAT^HA^) or β-tubulin (green) at the indicated stages of the parasite cell cycle. Images were merged with the DNA stain DAPI (blue). (C) IFAs of RH parasites stained for acetyl-K40–α-tubulin (red) or β-tubulin (green) at the indicated stages of the parasite cell cycle. Note that K40 acetylation is present on both spindle microtubules during mitosis and in the daughter subpellicular microtubules throughout replication. Scale bar, 3 μm.

To confirm the unusual size and assess the expression patterns of TgATAT, we introduced three C-terminal hemagglutinin (3×HA) epitope tags at the endogenous *TgATAT* locus by single-crossover homologous recombination in the RHΔ*ku80*Δ*hxgprt* parasites (50, 51). TgATAT^HA^ resolves as a single band migrating at the predicted size of 98 kDa, and the C-terminal 3×HA tag had no effect on K40 acetylation (Fig. 4A). IFAs show that TgATAT^HA^ exhibits cell cycle-regulated expression, with low levels appearing during S phase and mitosis and peak levels occurring during early cytokinesis (Fig. 4B). In agreement with the mRNA expression data (49), TgATAT^HA^ protein levels decrease as tachyzoites complete division and then remain undetectable throughout interphase (Fig. 4B). In agreement with other studies (35, 37), we observed that α-tubulin K40 acetylation occurs at both spindle and subpellicular microtubules during early daughter formation (Fig. 4C). Collectively, these findings suggest that TgATAT is expressed during the early stages of tachyzoite replication, when acetylation of nascent microtubule structures occurs.

### Disruption of *TgATAT* leads to loss of α-tubulin K40 acetylation.

**FIG 5:**
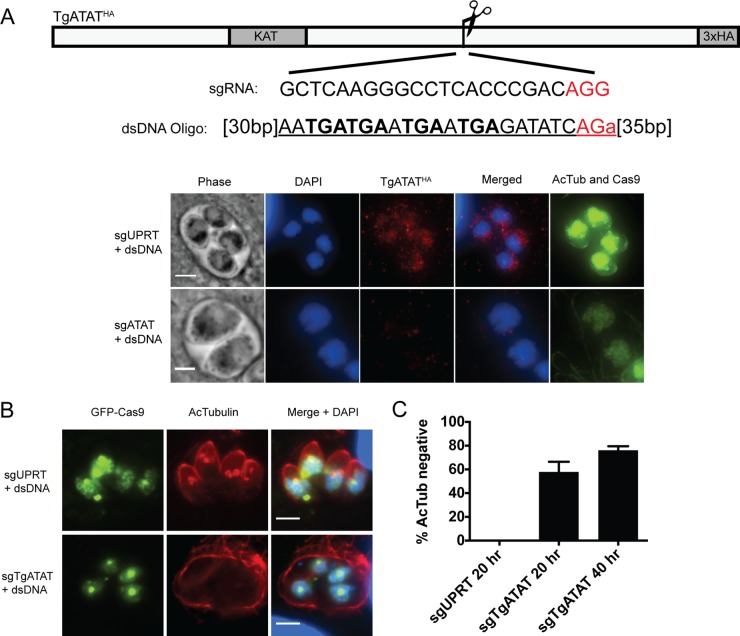
Selective targeting of GFP-Cas9 to the *TgATAT* locus eliminates K40 acetylation. (A) Diagram showing the site on *TgATAT* targeted by GFP-Cas9. The 20-bp *TgATAT* sgRNA sequence is shown; it is immediately upstream of the protospacer adjacent motif sequence (PAM, red). The dsDNA oligomer used for recombination is shown, and in brackets are the numbers of bases of homology flanking the PAM site. Underlined is the exogenous sequence introduced, including the four stop codons in bold. IFAs of dividing parasites expressing GFP-Cas9 confirm that disruption of *TgATAT* and loss of K40 acetylation occur only when Cas9 is targeted to the *TgATAT* locus (sgTgATAT). (B) IFA of TgATAT^HA^ parasites 40 h posttransfection with dsDNA oligomers and GFP-Cas9 targeted to either the *UPRT* (sgUPRT) or the *TgATAT* (sgTgATAT) locus. Scale bar, 3 μm. (C) Bar graph showing the percentage of GFP-Cas9-positive vacuoles that are acetyl-K40 negative in parasites in which GFP-Cas9 was targeted to *UPRT* (sgUPRT) versus *TgATAT* (sgTgATAT) 20 or 40 h after transfection. Error bars show the standard errors of the means (*n* = 3).

To determine the impact of TgATAT on α-tubulin K40 acetylation, we attempted to knock out the genomic locus by double homologous recombination but could not isolate viable clones, which suggests that TgATAT may be essential in tachyzoites. We then employed the CRISPR/Cas9 system recently reported for *Toxoplasma* (52, 53) by transfecting a plasmid encoding a green fluorescent protein (GFP)-Cas9 fusion and a *TgATAT*-targeting single guide RNA (sgRNA) into our TgATAT^HA^ parasites. Along with the GFP-Cas9/sgRNA plasmid, we cotransfected a double-stranded DNA (dsDNA) oligomer containing four stop codons flanked by short regions of homology to the *TgATAT* Cas9 cleavage site to ensure disruption of the gene, and the loss of TgATAT^HA^ protein was subsequently confirmed by IFA (Fig. 5A). A control transfection was performed with the aforementioned dsDNA oligomer and the GFP-Cas9 plasmid containing sequences encoding an sgRNA targeting the unrelated and nonessential uracil phosphoribosyltransferase (*UPRT*) gene (53). Transfected parasites were inoculated onto human foreskin fibroblasts (HFFs), and the infected monolayers were fixed after 20 h so that the expression of GFP-Cas9 could be observed by fluorescence microscopy (Fig. 5B). In parasites transfected with the *UPRT*-targeting construct, we observed 13% (30/231) expressing GFP-Cas9; in contrast, GFP-Cas9 expression was observed at a lower frequency in parasites transfected with *TgATAT*-targeting sgRNA (2.89% ± 0.93% [standard error of the mean], *n* = 3). Importantly, α-tubulin K40 acetylation was completely abolished in the majority of the GFP-Cas9-positive parasites transfected with the *TgATAT*-targeting sgRNA (57.8% ± 4.97%, *n* = 3) but never diminished in GFP-Cas9-positive parasites transfected with the control *UPRT*-targeting sgRNA (Fig. 5B and C). At 40 h posttransfection, the number of GFP-Cas9-positive parasites lacking α-tubulin K40 acetylation increased to 76.1% ± 2.01% (*n* = 3) in *TgATAT* sgRNA-transfected populations. The inability to detect α-tubulin K40 acetylation when the *TgATAT* locus is selectively targeted for CRISPR/Cas9-mediated disruption strongly suggests that TgATAT is the major α-tubulin K40 acetyltransferase in *Toxoplasma*.

### Loss of K40 acetylation causes microtubule defects and impairs replication.

**FIG 6:**
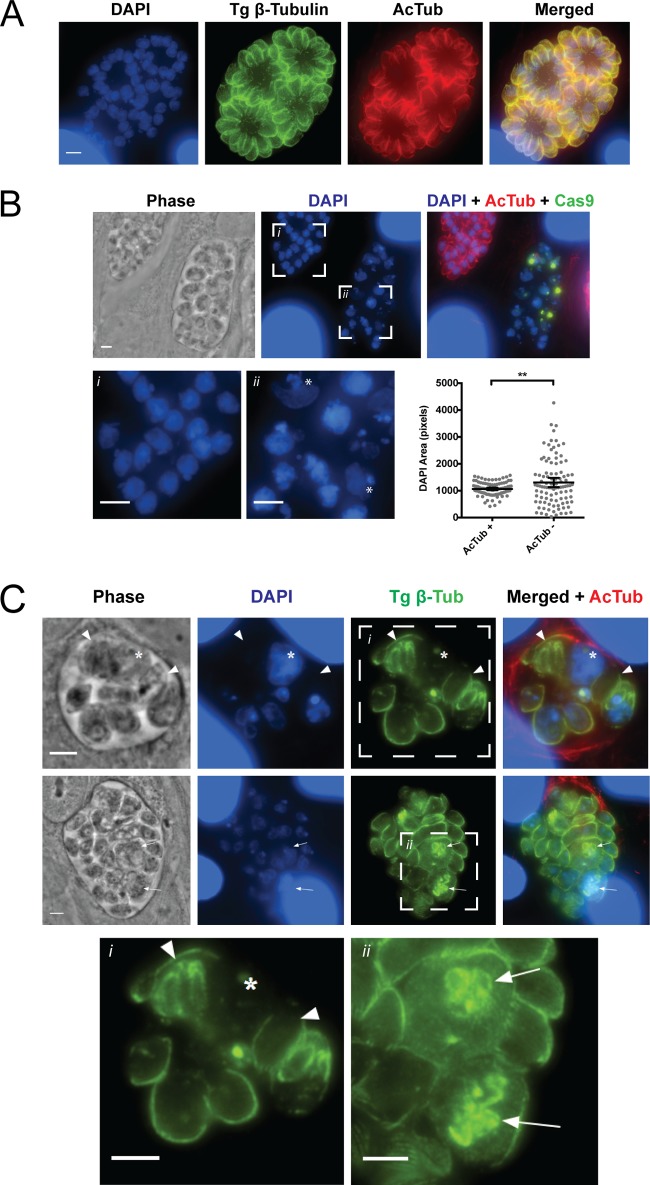
Defects in nuclear division and segregation in parasites lacking α-tubulin K40 acetylation. (A) TgATAT^HA^ parasites were transfected with GFP-Cas9-sgTgATAT and imaged at 40 h posttransfection. Shown are transfectants lacking GFP-Cas9 expression, which display normal replication and have microtubules containing K40 acetylation, as visualized with anti-β-tubulin (green) and acetyl-K40–α-tubulin (red) antibodies, with DAPI costain in blue. (B) Parasites expressing GFP-Cas9 lose K40 acetylation and contain abnormal nuclear morphology compared to that of parasites possessing K40 acetylation. Nuclei were visualized by DAPI staining (blue). Insets of acetyl-K40-positive (*i*) and -negative (*ii*) parasites are shown and expanded in the lower panels. The DAPI-stained structures was measured with ImageJ (*n* = 100 nuclei). Double asterisks indicate a significant difference in mean area, as determined by unpaired *t* test with Welch’s correction for unequal variance (P = 0.0085). (C) Vacuoles containing parasites lacking acetylated microtubules (red) and showing aberrant phenotypes detected by staining all of the microtubules (green) and DNA (blue) are shown. Inset i shows that parasites lacking K40 acetylation have defects in microtubule structures and fail to partition nuclear material into daughter parasites. Anucleate parasites are marked by arrowheads, while the improperly segregated nuclear mass is indicated by the asterisk. Inset ii shows parasites containing multiple β-tubulin structures resembling daughter cell conoids (arrows). Scale bar, 3 μm.

GFP-Cas9-expressing parasites transfected with the *UPRT* targeting control retained acetylated α-tubulin and displayed normal microtubule structures 40 h posttransfection, as did *TgATAT* sgRNA transfectants that lacked GFP-Cas9 expression (Fig. 6A). In contrast, *TgATAT* sgRNA transfectants expressing GFP-Cas9 that lost α-tubulin acetylation exhibited abnormal morphology (Fig. 6B, phase, compare acetyl-K40-positive parasites in inset i with acetyl-K40-negative parasites in inset ii). 4′,6-Diamidino-2-phenylindole (DAPI) staining revealed that the parasites lacking K40 acetylation possessed nuclei that were grossly deformed, as if they failed to complete karyokinesis (Fig. 6B, inset ii, asterisks). *TgATAT* sgRNA transfectants that retained acetylated α-tubulin contain nuclei that are uniform in shape and area (as measured in pixels), whereas parasites lacking acetylated α-tubulin possess a wide variety of DAPI-staining structures that are abnormal in size and shape (Fig. 6B). Of note, while large nuclei that appeared to have undergone genomic duplication were frequently observed, the classic horseshoe shape of the nucleus routinely seen during cytokinesis was largely absent from parasites lacking K40 acetylation. Further, many of the parasites lacking K40 acetylation contained daughter parasites that were devoid of nuclear material (Fig. 6C, inset i, arrowheads). Parasites lacking K40 acetylation were also observed to possess numerous additional cytoplasmic β-tubulin-containing structures, many of which resembled caps of budding daughter cells (Fig. 6C, inset ii, arrows); in these parasites, the mother’s microtubules and cytoskeleton fail to undergo recycling to the residual body and instead appear to remain intact while the formation of multiple daughter parasites seems to have been initiated. Despite this evidence of early daughter bud initiation, proper formation of the mature cytoskeleton appears to be impaired upon loss of K40 acetylation, as a variety of abnormal parasite sizes and shapes are evident (Fig. 6C, phase and β-tubulin channels). These phenotypes were not observed in sgUPRT/GFP-Cas9-positive parasites, nor were they seen in the subset of sgTgATAT/GFP-Cas9-positive parasites that retained tubulin acetylation, suggesting that the defects in nuclear morphology and parasite division are due to loss of K40 acetylation and not to Cas9 expression. Together, these defects in cytokinesis and cytoskeleton formation suggest that K40 acetylation likely impacts both spindle and subpellicular microtubule populations.

**FIG 7:**
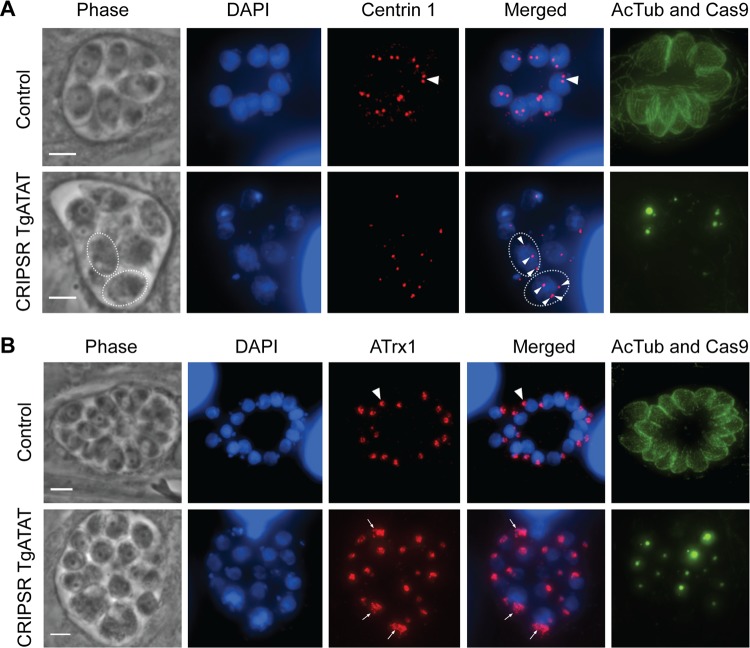
Centrosome duplication and apicoplast division in parasites lacking K40 acetylation. (A) Duplication of the centrosomes occurs in the presence (top row, arrowhead) or absence of K40 α-tubulin acetylation (bottom row, arrowheads), as visualized by IFA staining for centrin 1 (red), acetyl-K40–α-tubulin (green), and DNA (DAPI, blue). K40-acetylated α-tubulin and GFP-Cas9 (localized to the nucleus) were detected in the same channel (green). Note the loss of acetylated α-tubulin and GFP-Cas9-expressing parasites. Dotted lines encircle individual parasites with arrowheads indicating multiple centrosomes. (B) IFAs of parasites stained for apicoplast membrane protein Atrx1 (red), acetyl-K40–α-tubulin (green), DNA (DAPI, blue), and GFP-Cas9 (green, nuclear). Apicoplasts that underwent normal division are visible in acetyl-K40-positive parasites (top row, arrowhead). Apicoplasts that failed to divide in parasites lacking K40 acetylation (bottom row) are indicated by arrows. Scale bars, 3 μm.

To explore these replication defects more closely, we examined early steps in *Toxoplasma* endodyogeny when the duplication of the centrosome and subsequent division of the apicoplast occur (54). Control parasites containing K40 acetylation have either a single or a duplicated centrosome per nucleus (Fig. 7A, arrowhead), as well as a single apicoplast (Fig. 7B, arrowhead). Parasites lacking K40 acetylation can still duplicate centrosomes; however, those with defects in nuclear division often had >2 centrosomes per nucleus (Fig. 7A, arrows). Additionally, parasites lacking K40-acetylated α-tubulin often contained large, irregular apicoplasts, suggesting a role for acetylated α-tubulin in the proper division of this organelle (Fig. 7B, arrows).

**FIG 8:**
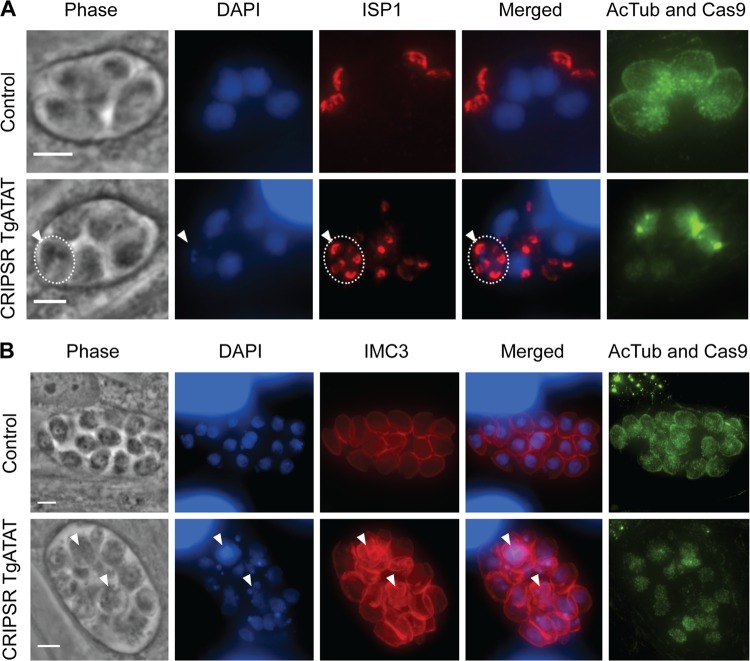
Daughter cytoskeleton completion is impaired upon loss of K40 acetylation. (A) IFA visualizing ISP1 (red), acetyl-K40–α-tubulin (green), DNA (DAPI, blue), and GFP-Cas9 (green, nuclear) in normal parasites (top) versus those lacking acetylated α-tubulin (bottom). Dotted lines encircle an individual parasites containing excess daughter buds (bottom row, arrowhead), as visualized by numerous ISP1-positive apical cap structures. (B) IFA stained for IMC3 (red), acetyl-K40–α-tubulin (green), DNA (DAPI, blue), and GFP-Cas9 (green, nuclear). Arrowheads indicate multiple IMC3-positive structures in parasites lacking K40 α-tubulin acetylation, indicative of partially formed daughter cells that failed to complete division. Scale bars, 3 μm.

Centrosome duplication is followed by formation of the daughter bud apical complex. As we observed evidence of apical conoid formation in the absence of K40 acetylation (Fig. 6C, arrowheads), we investigated whether K40 acetylation was required to recruit the IMC and IMC subcompartment proteins (ISPs) to the nascent daughter cytoskeleton. During interphase, ISP1 localizes to the apical cap of each parasite (Fig. 8A) and is recruited to the apical regions of the daughter buds during bud formation (55). While parasites containing acetylated α-tubulin showed two ISP1-labeled apical caps per dividing parasite, loss of α-tubulin acetylation resulted in parasites containing multiple ISP1 structures per nucleus (Fig. 8A, arrowhead). In parasites lacking acetylated α-tubulin, IFAs staining for IMC3, which localizes to the IMC following recruitment of ISP1 to daughter caps, revealed the presence of multiple IMC structures forming within a single parasite (Fig. 8B, arrowheads). Taken together, these results suggest that acetylation of α-tubulin at K40 is dispensable for initiation of mitosis and the early steps of daughter cell formation but is required for karyokinesis, apicoplast division, and completion of *Toxoplasma* replication.

## DISCUSSION

In this study, we addressed the biological role of α-tubulin K40 acetylation in the protozoan pathogen *Toxoplasma* by two independent genetic approaches that ablated either the modifying enzyme or the substrate. Our mutational analyses of *TgTUBA1* show that mutation of K40 to glutamine, an acetyl-lysine mimic, is tolerated, while mutation to arginine is inviable unless a secondary mutation that stabilizes microtubules is made. Additionally, we have identified and characterized the KAT responsible for mediating K40 acetylation, TgATAT, and have shown that loss of K40 acetylation following CRISPR/Cas9-mediated disruption of the *TgATAT* locus severely impairs nuclear division and parasite replication.

Interestingly, many of the phenotypes we observed upon the disruption of K40 α-tubulin acetylation parallel the effects observed when microtubules are destabilized with oryzalin (13, 14, 39, 56, 57). Treatment of intracellular parasites with oryzalin prevents completion of the daughter budding process, although nuclear size increased and centriole replication occurred (41). Additional studies show that destabilization of the spindle microtubules by oryzalin results in the formation of anuclear “zoids,” similar to what we observed in parasites lacking K40 acetylation (Fig. 6C) (14). Importantly, electron microscopic analysis of oryzalin-treated parasites showed that numerous daughter conoids formed within mother parasites when nuclear division failed. This bears a striking resemblance to the defects we observed in parasites lacking K40 acetylation, as we detect numerous structures resembling daughter caps that stain positive for β-tubulin, ISP1, and IMC3 (Fig. 6C and 8A and B). Early stages of parasite division, including replication of genomic DNA, duplication of the centrosomes, and daughter bud initiation, remain intact following loss of K40 acetylation, again mirroring what is seen in oryzalin-treated parasites. In addition to defects in nuclear division, we also saw evidence that apicoplast division is impaired in parasites without K40 acetylation (Fig. 7B, arrows). This agrees with studies showing that apicoplast division is a microtubule-dependent process that occurs temporally with nuclear division and is disrupted by oryzalin treatment (58). Together, these results suggest that acetylation of α-tubulin at K40 plays an important role in stabilizing both the subpellicular and mitotic spindle populations of microtubules in *Toxoplasma* and that this microtubule stabilization is especially critical for proper tachyzoite replication.

Our mutational analyses of *TgTUBA1* showed that the function of K40 acetylation can be mimicked by replacement of K40 with glutamine (Fig. 1), in agreement with findings in both *C. elegans* and *Rattus norvegicus* (25, 59). Molecular dynamic simulations provide a plausible explanation for the ability of the K40Q change to effectively mimic K40 acetylation. In those simulations, acetylation of K40 on α-tubulin disrupts an intramonomeric salt bridge between α-tubulin residues K40 and E55, a residue conserved in *Toxoplasma* α-tubulin. This disruption allows the acetyl group on K40 to form a new salt bridge with H283 (also conserved in *Toxoplasma*) of an α-tubulin monomer in an adjacent protofilament (30). Mutation of K40 to arginine would chemically allow salt bridge formation with the carboxyl group of E55 while preventing interprotofilament salt bridge formation through H283. Conversely, mutation of K40 to glutamine would constitutively promote salt bridge formation between the glutamine carboxyl group (Q40) and the imidazole group (H283) located on the adjacent protofilament. Therefore, our ability to generate parasites expressing only Q40 in the nonstabilizing V252L background suggests that salt bridge formation between protofilaments is important for microtubule structure and stability.

The ability to generate K40R mutants in the T239I background further speaks to the role of K40 acetylation in *Toxoplasma* microtubule stability. T239I resides in the H7 helix of α-tubulin and is within the oryzalin binding pocket (60). Mutation of another binding pocket residue (L136F) stabilized microtubules by promoting interactions between α-tubulin subunits within the microtubule lattice (41). Together, these data suggest that oryzalin resistance generated by the mutation of binding pocket residues may also lead to increased interprotofilament interactions, which is similar to the proposed mechanism by which K40 acetylation promotes microtubule stability discussed above. Therefore, it is plausible that, like L136F, the T239I mutation stabilizes microtubules and compensates for the loss of stability when K40 acetylation is nullified.

To further determine the biological importance of α-tubulin K40 acetylation in *Toxoplasma*, we identified and disrupted the α-tubulin acetyltransferase gene *TgATAT*. Endogenous tagging of the *TgATAT* locus revealed a cell cycle expression pattern that correlated temporally with enriched regions of α-tubulin acetylation during parasite replication. These results agree with a recent report noting that α-tubulin K40 acetylation occurs after spindle microtubule assembly in early daughter formation (37). TgATAT is considerably larger than all previously characterized ATAT/Mec-17 proteins, possessing highly divergent sequences flanking the conserved Mec-17 domain (Fig. 3A). Further bioinformatic analysis revealed that selected species within the coccidian subclass of the *Apicomplexa* phylum also possess a large TgATAT homologue, 89 kDa in *Neospora caninum* (NCLIV_010560) and 98 kDa in *Hammondia hammondia* (HHA_319600). Our CRISPR-mediated disruption of *TgATAT* indicates that it is the primary α-tubulin acetyltransferase in *Toxoplasma*.

While mammalian ATAT has been shown to localize to the lumen of the microtubule, where it exerts its KAT activity (61), the considerably larger size of the apicomplexan orthologues may seem to preclude their access to this site. However, recent *in vitro* studies using polymerized microtubules have shown that large macromolecules, such as the full-length 6-11B-1 anti-acetyl-K40–α-tubulin antibody (~150 kDa), are able to enter the lumen of microtubules (61). Other studies have also reported that ATAT/Mec-17 can bind to the exterior surface of microtubules; if microtubules undergo lateral opening between protofilaments, the K40 substrate could be exposed for acetylation (62, 63). Further studies are required to address exactly how TgATAT gains access to its substrate. Whether TgATAT has additional substrates is also an intriguing question for future study. Besides ATAT/Mec-17 itself (64), no other substrates have been reported for this family of KATs to date.

Our analysis of K40 acetylation provides the first detailed look at the functional role and importance of tubulin modifications in *Toxoplasma* biology. Given that many other tubulin PTMs have been reported for *Toxoplasma* (34), it is likely that these modifications may also contribute to the regulation of microtubule function in the parasite. While dissecting the biological role of these PTMs in other species is complicated by the expression of multiple α-tubulin isotypes, their function in *Toxoplasma* tachyzoites may be easier to determine, as only *TgTUBA1* is expressed in this stage. The identification and characterization of the enzymes responsible for mediating these PTMs provide an exciting new field of study for the development of novel therapeutics to treat toxoplasmosis.

## MATERIALS AND METHODS

### Antibodies.

The following primary antibodies were used at the dilutions indicated: rabbit anti-*T. gondii* β-tubulin (1:2,000 [14]), mouse anti-acetyl-K40–α-tubulin (1:2,000; Sigma 6-11-B-1), rabbit anti-acetyl-K40–α-tubulin (1:2,000; EMD Millipore ABT241), mouse anti-SAG1 (1:2,000; Genway), rat anti-IMC3 (1:2,000 [65]), rat anti-ISP1 (1:1,000 [55]), rabbit anti-centrin 1 (1:1,000 [66]), mouse anti-Atrx1 (1:2,000; 11G8 [67]), and rat anti-HA (1:1,000; Roche). The secondary antibodies used for immunoblot assays included donkey anti-rabbit (1:2,000; GE Healthcare), sheep anti-mouse (1:5,000; GE Healthcare), and goat anti-rat (1:2,000; GE Healthcare) antibodies conjugated to horseradish peroxidase. Fluorophore-conjugated secondary antibodies (Alexa Fluor; Thermo Fisher) were used for IFAs at a 1:2,000 dilution (1:1,000 for TgATAT^HA^).

### Parasite culture and transfection.

*Toxoplasma* parasites were maintained in HFFs with Dulbecco’s modified Eagle’s medium supplemented with 10% heat-inactivated fetal bovine serum at 37°C and 5% CO_2_ (68). To assess parasite doubling time, purified tachyzoites were allowed to invade HFF monolayers in 12-well plates for 2 h. Extracellular parasites were removed, and infected monolayers were incubated for an additional 22 h before being fixed in methanol for staining with a Differential Quik Stain kit (Polysciences, Inc.). For generation of TgTUBA1 K40 mutants, wild-type RH parasites were electroporated with 100 µg of linearized plasmid and selection medium containing 2.5 µM oryzalin was added 24 h following transfection. Similarly, TgATAT^HA^ parasites were generated by transfection of RHΔ*hx*Δ*Ku80* parasites (50, 51) with 75 µg of linearized plasmid and selected with medium containing 1 µM pyrimethamine. Following three passages under drug selection, resistant parasites were cloned by limiting dilution in 96-well plates.

### Generation of TgTUBA1 K40 mutant parasites.

The plasmid construct described in reference 40 containing the *TgTUBA1* genomic sequence carrying the T239I oryzalin resistance mutation was used as the template for site-directed mutagenesis with the QuikChange II kit (Agilent) to introduce K40K silent, K40R, and K40Q mutations with primers 1 to 6 (see Table S1 in the supplemental material). This construct was then linearized by digestion with NotI-HF and HindIII-HF and transfected into RH strain parasites by electroporation as described above. Transfected parasites were selected by growth in medium containing 2.5 µM oryzalin, and surviving parasites were cloned by limiting dilution into 96-well plates. For the generation of V252L mutant strains, T239I was restored to T239T, the V252L mutation was introduced with the Q5 Site-Directed Mutagenesis kit (New England Biolabs) with primers 7 to 10, and stable clones were selected as described above. Single clones were confirmed by PCR amplification of the *TgTUBA1* locus from genomic DNA purified with the DNeasy Blood and Tissue kit (Qiagen) and sequencing with primers 11 and 12.

10.1128/mSphere.00088-15.1 TABLE S1  Primers used in this study. Download TABLE S1, DOCX file, 0.01 MB. Copyright © 2016 Varberg et al. 2016 Varberg et al. This content is distributed under the terms of the Creative Commons Attribution 4.0 International license.

### Endogenous tagging of TgATAT.

Genomic DNA was isolated from RHΔ*hx*Δ*ku80* parasites, and primers 13 and 14 (see Table S1 in the supplemental material) were used to amplify ~1.2 kb of the *TgATAT* gene just upstream of the stop codon. The in-Fusion HD cloning kit (Clontech) was used to clone the amplified *TgATAT* sequence into the PacI restriction site of pLIC.HA3.DHFR (69). Prior to transfection, 75 µg of plasmid DNA was linearized by NcoI and electroporated into RHΔ*hx*Δ*ku80* parasites. Following three passages under selection with 1.0 µM pyrimethamine, parasites were cloned by limiting dilution and positive clones were identified by immunoblotting.

### Immunoblotting.

Freshly egressed parasites were passed through 3.0-μm filters to remove host cell debris and then washed in phosphate-buffered saline (PBS). Parasites were lysed in radioimmunoprecipitation assay buffer supplemented with complete protease inhibitor cocktail (Roche), sonicated twice for 10 s each time with a microtip sonicator, and centrifuged to remove insoluble debris. Cleared lysate was subjected to SDS-PAGE with precast 4 to 20% Mini-PROTEAN TGX gels (Bio-Rad), and resolved proteins were transferred to nitrocellulose membranes with the Transblot SD semidry transfer system (Bio-Rad). Membranes were blocked in 5% milk–Tris-buffered saline–Tween 20 (TBST) and probed with primary antibodies for 1.5 h at room temperature or overnight at 4°C. Membranes were washed three times for 10 min each time in TBST and probed with secondary antibodies for 45 min at room temperature. Membranes were washed again three times for 10 min each time in TBST, and proteins were detected with SuperSignal West Femto substrate (Thermo Fisher) and imaged on a FluorChem R imager (Bio-Techne).

### IFAs.

Confluent HFF monolayers grown on coverslips in 24-well plates were infected with freshly lysed parasites. For all images of microtubules, cells were fixed with cold methanol, washed with PBS, and blocked for 30 min with 3% BSA in PBS. For imaging of TgATAT^HA^, cells were fixed with 4% paraformaldehyde in PBS, blocked for 30 min in 3% BSA–PBS, and permeabilized with PBS with 0.2% Triton X-100 (PBS-T) for 10 min. Primary antibodies diluted in 3% BSA–PBS were applied for 1.5 h at room temperature or (for TgATAT^HA^) overnight at 4°C in BSA–PBS-T. Coverslips were washed three times for 15 min each time with PBS, and secondary antibodies were added in 3% BSA–PBS for 45 min. Following three 15-min washes in PBS, coverslips were mounted with Vectashield antifade mounting medium (Vector Labs) containing DAPI to visualize nuclei.

### CRISPR-mediated disruption of *TgATAT*.

The plasmid described in reference 53 (GFP-Cas9/sgUPRT) was used for site-directed mutagenesis with the Q5 kit to delete the HA epitope tag with primers 15 and 16 and to mutate the sgRNA sequence to target the *TgATAT* locus with primers 17 and 18. TgATAT^HA^ parasites were transfected with 50 µg of the purified *TgATAT*- or *UPRT*-targeted GFP-Cas9 plasmid and 50 µg of 90-bp dsDNA oligonucleotides 19 and 20. Transfected populations were used to inoculate coverslips and were fixed at the time points indicated in the figures.
