# Supplementary material for: TgATAT-Mediated α-Tubulin Acetylation Is Required for Division of the Protozoan Parasite Toxoplasma gondii
Source: mSphere. 2016 Jan 20;1(1):e00088-15. doi: 10.1128/mSphere.00088-15 (PMC4863603; doi:10.1128/mSphere.00088-15)
Supplement: TABLE S1 [file sph001162010st1.docx]

**Supplemental Table 1: Primers used in this study.**

|  | # | Name | Primer Sequence Used |
| --- | --- | --- | --- |
| *TUBA1*  Site-directed | 1 | K40K F | GCAGATGCCCTCTGACAAAACCATTGGAGGTGG |
|  | 2 | K40K R | CCACCTCCAATGGTTTTGTCAGAGGGCATCTGC |
|  | 3 | K40R F | GCAGATGCCCTCTGACCGCACCATTGGAGGTGG |
|  | 4 | K40R R | CCACCTCCAATGGTGCGGTCAGAGGGCATCTGC |
|  | 5 | K40Q F | GCAGATGCCCTCTGACCAGACCATTGGAGGTGG |
|  | 6 | K40Q R | CCACCTCCAATGGTCTGGTCAGAGGGCATCTGC |
| Oryzalin Mutation | 7 | I239T F | gagagacgcggtcagggaggagatgacctg |
|  | 8 | I239T R | caggtcatctcctccctgaccgcgtctctc |
|  | 9 | V252L F | cgctcaacgtcgacttgactgagttccagac |
|  | 10 | V252L R | gtctggaactcagtcaagtcgacgttgagcg |
| *TgTUBA1* Sequencing | 11 | *TgTUBA1* F | ATGAGAGAGGTTATCAGCATC |
|  | 12 | *TgTUBA1* R | TTAGTACTCGTCACCATAGCC |
| *TgATAT* tagging | 13 | *TgATAT*_F | ttccaatccaatttaATTTCTACGTCCTCGAGAGCTGT |
|  | 14 | *TgATAT*_R | ccacttccaattttaaaCGACCAGTTGAGGAGAGACG |
| Deletion of HA tag in GFP-Cas9/sgUPRT | 15 | CRISPR HA del F | AGCCTGGGCAGCGGCTCC |
|  | 16 | CRISPR HA del R | GGCGTCGCCTCCCAGCTG |
| *TgATAT* CRISPR sgRNA | 17 | *TgATAT* sgRNA F | ctcacccgacGTTTTAGAGCTAGAAATAGCAAG |
|  | 18 | *TgATAT* sgRNA R | gcccttgagcAACTTGACATCCCCATTTAC |
| *TgATAT* CRISPR Oligos | 19 | *TgATAT* Oligo F | tccactccgagctcaagggcctcacccgacAATGATGAATGAATGAGATATCagAccgccttccaccggctcctgccgacgctccagtcg |
|  | 20 | *TgATAT* Oligo R | cgactggagcgtcggcaggagccggtggaaggcggTctGATATCTCATTCATTCATCATTgtcgggtgaggcccttgagctcggagtgga |
